# Relationship of cell surface hydrophobicity with biofilm formation and growth rate: A study on *Pseudomonas aeruginosa, Staphylococcus aureus, *and* Escherichia coli*

**DOI:** 10.22038/IJBMS.2018.28525.6917

**Published:** 2018-07

**Authors:** Zulfiqar Ali Mirani, Aiman Fatima, Shaista Urooj, Mubashir Aziz, Mohammad Naseem Khan, Tanveer Abbas

**Affiliations:** 1 Microbiology Analytical Centre, PCSIR Laboratories Complex Karachi, Pakistan; 2 Department of Microbiology, University of Karachi, Pakistan; 3 Department of Microbiology, Federal Urdu University of Arts, Science, and Technology, Pakistan; 4 Department of Pathobiology, Bahauddin Zakariya University, Multan, Pakistan

**Keywords:** Biofilms, Escherichia coli, Hydrophobicity, Pseudomonas aeruginosa, Small colony variants, Staphylococcus aureus

## Abstract

**Objective(s):**

This study was designed to determine the relationship of *Pseudomonas aeruginosa, Staphylococcus aureus, *and* Escherichia coli* isolates in multispecies biofilms and their individual phenotypic characters in biofilm consortia.

**Materials and Methods:**

The subject isolates were recovered from different food samples and identified on the basis of growth on differential and selective media. Tube methods, Congo-red agar method, and scanning electron microscopy (SEM) were used to study biofilms phenotypes. The hydrophobicity of the strains was evaluated by the adhesion to apolar solvent.

**Results:**

The results showed that *E. coli *dominated the pre-biofilm stage. It has been observed that *E. coli* adopted biofilm life much before *S. aureus *and* P. aeruginosa*. However, after adopting biofilm lifestyle, slowly and gradually, *P. aeruginosa* dominated the consortia and dispersed other stakeholders. The subject isolates of *P. aeruginosa* produce cis-2-decanoic acid to disperse or inhibit *S. aureus *and* E. coli* biofilms. Gas-chromatography and mass spectrometry results showed that cis-2-decanoic was higher in the co-culture condition and increased at late log-phase or at stationary phase. Although majority of *S. aureus *were unable to compete with *P. aeruginosa*, however, a minor population competed, survived, and persisted in biofilm consortia as small colony variants. The survivors showed higher expression of sigB and sarA genes. *P. aeruginosa* showed comparatively higher hydrophobic surface properties.

**Conclusion:**

Comparative analysis showed that cell surface hydrophobicity, growth rate, and small colony variants (SCVs) are correlated in biofilm consortia of the *P. aeruginosa, S. aureus, *and* E. coli.*

## Introduction

In the natural environment, bacteria exhibit remarkably diverse and complex social cooperation and coordination. Majority of bacteria have the ability to switch from unicellular to multicellular lifestyle such as microbial colonies and biofilms (1, 2). The biofilms are bacterial aggregates adherent to each other and/or to a surface embedded in self-produced extracellular polymeric substances (3). These highly organized communities comprise a high level of morphotypes and phenotypic and genotypic heterogeneity where inter- and intra-species interaction and cooperation is a common phenomenon (4). In biofilm consortia, bacteria suspend their metabolism and cover themselves in a shell made of polysaccharides and proteins (5), which protects biofilm indwellers from the toxic effects of antibacterial agents. Most of the antibiotics target actively growing bacteria and are unable to penetrate protective layer of biofilms (5). In biofilm structure, coordination and cooperation take place via inter and intraspecies exchange of metabolites, signaling molecules, genetic material, and defensive compounds (4). More often diffusible signal molecules or cell-density related (quorum sensing - QS) molecules are released to communicate and induce expression of certain genes in neighboring cells (6). According to Atkinson and Williams (7), bacteria are not limited to communication within their own species but are capable of ‘listening in’ and ‘broadcasting to’ unrelated species to intercept messages and coerce cohabitants into behavioral modifications, either for the good of the population or for the benefit of one species over another. It is highly evident that several species of bacteria coexist and interact in multispecies biofilm consortia and all of the indwellers coordinate for survival (8). In settings where multiple species coexist, their interactions often are mediated through extracellular compounds (4). Development in one microbe can be influenced by small molecules secreted by other species (9). As the organisms adhere to a surface, they keep signaling to one another. Once they sense a quorum, genes are up-regulated and sticky exo-polysaccharides are produced that ‘glue’ the bacteria together (4, 5). It has been reported that *Escherichia coli* interacts with other bacteria, e.g. pseudomonas and staphylococci and is able to form multi-species biofilms (5). Similarly, *Pseudomonas aeruginosa* PAO1 has been reported to facilitate *Staphylococcus aureus* biofilm formation when both are grown in coculture biofilms (10). In the process, eDNA facilitates interspecies interaction between *P. aeruginosa *and *S. aureus* (4, 11). It has also been reported that exoproducts of* P. aeruginosa* recovered from cystic fibrosis patients stimulate *S. aureus* biofilms (12). Similarly, peptidoglycan shed by *S. aureus* was found to stimulate the production of virulence factors in *P. aeruginosa* (12, 13)*. *Likewise, *P. aeruginosa* can greatly increase the ability of *E. coli* to persist and grow in aquatic environments as well as in biofilm formation (14). These three pathogens *S. aureus, P. aeruginosa, *and *E. coli *are well known for their versatility and pathogenicity. The study of physiology and behavior of multispecies biofilm of foodborne pathogens in food processing environments may provide the necessary information to prevent and reduce the contamination of food products. Therefore, it is necessary to understand the inter-species and intra-species interactions, i.e. population structure, cell surface charges, physiology, and function within a biofilm. Although, *S. aureus, P. aeruginosa, *and *E. coli *are associated with the hospital as well as community-acquired infections, to the best of our knowledge, nothing is yet known about the formation of a mixed-species biofilm composed of these pathogens. The aim of this study was to characterize the inter-species interaction of these pathogens in biofilm consortia.

## Materials and Methods

In the present study, a total of seventeen biofilm-producing and five non-biofilm-producing strains of *P. aeruginosa*, *E. coli,* and *S. aureus* were used.   The subject strains were isolated from different food samples. These isolates were identified on the basis of typical morphology by gram staining and growth on differential and selective media, e.g. Cetrimide Agar (Merck, Germany) for *P. aeruginosa*, Eosin methylene blue *(*EMB, Oxoid) and MacConkey agar (Oxoid) for* E. coli, *and Baird*–*Parker agar (Oxoid) with egg yolk-tellurite (Sigma) for* S. aureus. *Coagulase and DNase tests were used for confirmation of *S. aureus *and oxidase test was performed for confirmation of* P. aeruginosa *and* E. coli.* *E. coli* (ATCC8739), *S. aureus* (ATCC25923), and *P. aeruginosa* (ATCC9027) were used as positive control for identification.

***Phenotypic characterization of slime-producing bacteria***

Biofilm formation was initially confirmed by the Congo red agar method as described earlier (15-17). Briefly, BHI agar (Oxoid) plates containing 50 g/l sucrose and 0.8 g/l Congo red were prepared and streaked with strains and incubated aerobically for 24–48 hr at 37 ^°^C. Positive results were indicated by black colonies with dry crystalline appearance. Weak slime producers usually remained pink, though occasional darkening at the center of colonies was observed.

***Biofilm assay***

A qualitative assessment of biofilm formation on glass slides was evaluated as described earlier by Mirani and Jamil (18). As it was difficult to study all of the seventeen isolates in different combinations, the isolates were randomly selected by using Microsoft Excel random number (RAND) generator from the group of subject isolates of this study. Briefly, two-inch pieces of glass slides were submerged in BHI broth (Oxoid) containing 0.1 ml of the 4 hr young culture of subject isolates of *P. aeruginosa*, *E. coli,* and *S. aureus *in a combination of randomly selected isolates and incubated at 37 °C for 48 hr to 96 hr.

*P. aeruginosa *+ *E. coli* + *S. aureus*

*P. aeruginosa *+ *E. coli*

*P. aeruginosa *+* S. aureus*

*E. coli* + *S. aureus*

All isolates in pure form

After incubation, glass slides were washed with phosphate buffer saline (pH 7.0) to remove unbound cells and debris; films were fixed with acetic acid for 15 min and stained with 3% crystal violet. Each experiment was repeated three times.

***Quantification of biofilms***

**Biofilm formation was quantified by the addition of 2 × 200 μl of 95% ethanol as described previously **(19) and absorbance was recorded with a spectrophotometer (Nicollet Evolution 300 BB) at 563 nm wavelength.

***Enumeration of biofilm population***

After maturation of the biofilm, the glass slides (4 mm) were gently washed three times with phosphate-buffered saline (PBS) to remove debris. After washing, glass slides were transferred to sterile 5 ml tubes containing 3 ml PBS and vortexed at 3000 rpm for 2 min to separate cells from biofilms. After vortexing, the extracted bacteria were enumerated using agar dilution plating technique. To perform it, 10 fold serial dilutions (1/10, 1/100, and 1/1000) were made from each sample containing the dislodged bacteria and 10 ml were seeded to calculate an accurate count of the biofilm population. Each experiment was performed in triplicate.

***Evaluation of colony variance during biofilm*** ***development and detection of persister cells***

The emergence of colony variants associated with biofilms of subject isolates was studied and these variants were enumerated, as described in a previous study (20). Biofilm biomass was harvested from a glass slide, resuspended in saline (total volume of 1 ml), homogenized for 30 sec to disrupt cell clusters by vigorous shaking, serially diluted, and plated on tryptone soy agar (Oxoid) and Baird–Parker agar (Oxoid), Cetrimide agar, and EMB agar plates. For the determination of stability of the colony variants, well-isolated colonies were sub-cultured on tryptone soy agar and incubated for 24 hr. This was repeated six times, and reversion with respect to colony size and biochemical reactions was monitored as described elsewhere (21). The experiments were performed in duplicate. The persister cells obtained were characterized for stability, oxidase, hemolysis, catalase production, clumping factor, coagulase production, and DNase production by the method of Bayston *et al* (22). The drop plate method described by researchers (23) was followed to count CFUs. 

***Scanning electron microscopy (SEM)***

Scanning electron microscopy (SEM) was done to analyze the production of extracellular matrix material and cell morphology as described earlier (18). Biofilm slides were divided into 4 mm sections and washed with distilled water to remove debris, and negatively stained with 0.2% uranyl acetate for 30 sec. These 4 mm slide sections showed the presence of biofilm material when examined directly in a GOEL-JEM-1200 EX II Electron Microscope.

***Bacterial hydrophobicity assay***

The hydrophobicity of strains was evaluated by the microbial adhesion to solvent test as described in a study (24). It consisted of evaluating the affinity of the cells towards apolar solvents (hexadecane). For the experiment, bacterial cells were harvested by centrifugation at 8500 g for 5 min and resuspended to ABS 578 nm in 0.01 M potassium phosphate buffer (pH 7.0). This bacterial suspension was mixed with a solvent in a ratio of 1:6 (0.4/2.4 v/v) by vortexing for 3 min to make an emulsion. The mixture was then left for 30 min until the separation of the two phases. Aqueous phase absorbance was measured (ABS 2) and the percentage of adhesion was expressed as %adhesion= (1–ABS 2/ ABS 1) × 100.

***Extracellular fatty acid (Cis-2-decenoic acid) extraction and methylation***

Fatty acids extraction was achieved by a method as described in a study (25). 50-ml samples of cell cultures of different groups were collected at different time intervals and centrifuged at 10,000 rpm for 15 min to remove cells and debris to collect the cell-free supernatant. The extracellular fatty acid in the supernatant was extracted with 50 ml of chloroform/ methanol mixture (2:1, v/v). The cell pellets were re-suspended in 10 ml distilled water and mixed with 20 mL chloroform/ methanol mixture (2:1, v/v). Cis-2-decenoic acid 100 μl/l was added as the internal standard. The chloroform layer was evaporated under a nitrogen stream and the dried fatty acid sample was used for the analysis of extracellular fatty acid. Separated fatty acids were methylated to prepare fatty acid methyl esters as described by Mirani and Jamil (26).

GC-MS analysis The GC-MS analysis for fatty acid methyl esters was performed as described previously by Mirani and Jamil (26) on an Agilent 6890 N Gas Chromatographic instrument coupled with an Agilent MS-5975 inert XL Mass Selective Detector and an Agilent auto-sampler 7683-B Injector (Agilent technologies, Little Fall, NY, USA). A capillary column HP-5MS (5% phenyl methylsiloxane) with a dimension of 60 m x 0.25 mm i.d × 0.25 µm film thickness (Agilent technologies, Palo, Alto, CA, USA) was used for the separation of fatty acid methyl esters. The initial temperature was 150 ºC, which was maintained for 2 min, raised to 240 ºC at the rate of 3 ºC/min and kept at 230 ºC for 10 min**. **The split ratio was 1:50 and helium was used as a carrier gas with the flow rate of 0.8 ml/min. The injector and detector were 240 and 270 ºC, respectively. The mass spectrometer was operated in the electron impact (EI) mode at 70 eV and scan range of 50–800 m/z. Library search identification of compounds from mass spectra was optimized and tested by matching test spectra against reference spectra in the NIST mass spectral database. Each experiment was repeated three times.

***Calculation and standard analysis***

Peak identification of analyzed extracellular fatty acids was covered by the comparisons with retention time and mass-spectra of known standards. Standard methyl esters of Cis-2-decenoic acid (100 μl/l) were used for the confirmation of GC-MS library results. All samples were used in duplicate, analyzed three times, and reported as n=2×3.

***Polymerase chain reaction***

The SCC*mec* elements (I–V) and *mecA* gene were identified as previously described (27, 28). Expression of *icaA,* *sigB,* and *sarA* genes was studied by using specific primers reported earlier (29-31). Total RNA was recovered from exponentially growing cells in tryptone soy broth (OD at 578 nm) using a dedicated kit (Qiagen Rneasy Mini, Qiagen, Hilden, Germany) and stored at −20 ^°^C. DNA was removed from RNA extractions using DNase according to the manufacturer’s instructions and RNA concentration was quantified using a spectrophotometer (Evolution 300 BB, Thermoelectro Corporation, Madison, WI, USA). One µg of RNA was used per reverse transcriptase-PCR (One-step RT-PCR Kit, Qiagen) together with gene-specific primers. Moreover, *P. aeruginosa* and *E. coli* were reconfirmed by using specific primers targeting 16s ribosomal DNA (rDNA) and *fimA*, respectively, as described by Spilker *et al. *(32) and Naravaneni and Jamil (33) (supplement 1). *E. coli* (ATCC8739), *S. aureus* (ATCC25923) and *P. aeruginosa* (ATCC9027) were used as positive control for identification. 

***Statistical analysis***

Data analysis was accomplished by using SPSS version 17.0 for frequencies. Data presented in result tables are mean of three independent experiments. 

## Results

***Co-culture of biofilm positive P. aeruginosa, S. aureus and E. coli***

**Figure 1:**
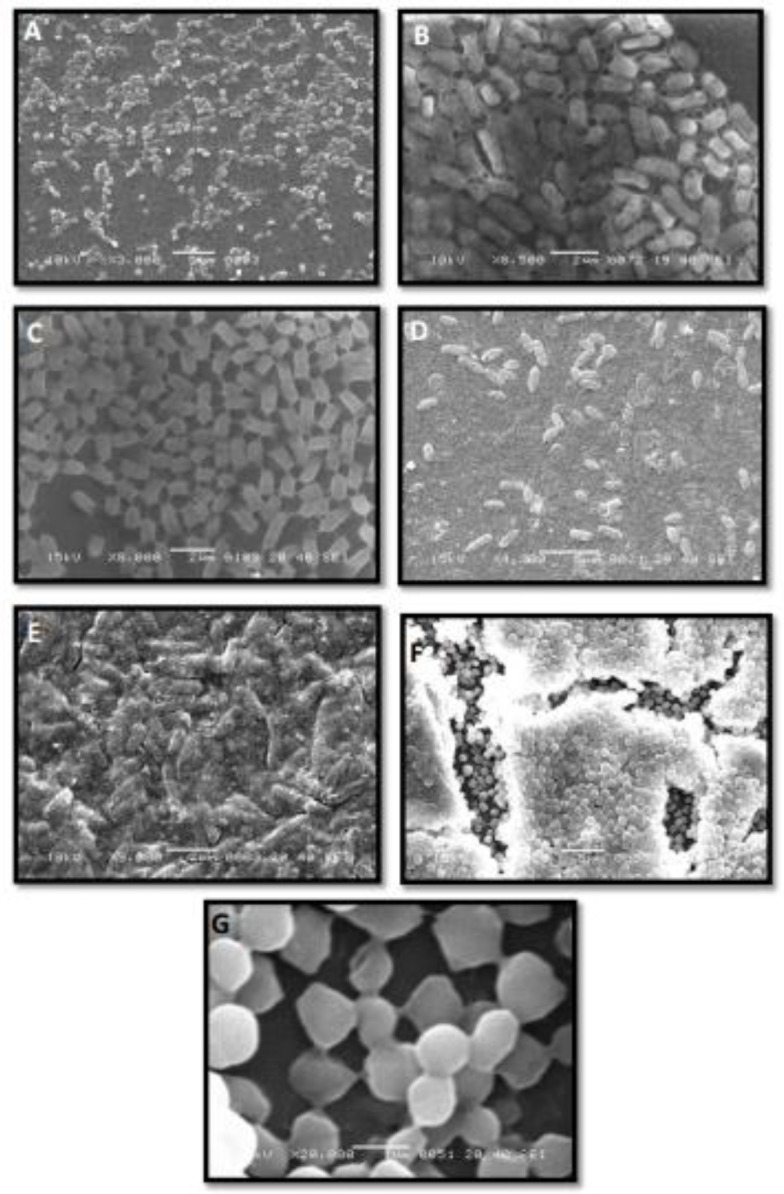
(A) *Pseudomonas aeruginosa* biofilm negative control, (B) *P. aeruginosa* biofilms, cells surrounded with the extracellular matrix material and showing brick-like appearance. (C) Highly adherent small colony variants of *P. aeruginosa*. (D) Biofilm negative *Escherichia coli.* (E) Biofilm consortia of *E. coli. *(F) Biofilm consortia of *Staphylococcus aureus*, cells surrounded with matrix material, showing multilayered structure. (F) Highly adherent small colony variants of *S. aureus*

*P. aeruginosa,* *S. aureus,* and *E. coli* are among the most prevalent foodborne pathogens. This study was designed to investigate the interspecies interactions of these pathogens in multi-species biofilms. For this study, seventeen biofilm positive isolates of each of* P. aeruginosa,* *S. aureus,* and *E. coli *were cocultured in TSB broth in different combinations. Initially, the individual characteristics of these isolates were studied in mono-species biofilm consortia. After adaptation to biofilm mode of life, all of these isolates were found to be more hydrophobic as compared to planktonic or wild-type isolates and cell surface hydrophobicity increased with incubation time (Table 1). This is a common character of biofilm positive isolates studied. Comparative analysis showed that *P. aeruginosa* possessed more hydrophobic surfaces among three subject isolates studied. Similarly, *S. aureus* showed more hydrophobic surface properties as compared to *E. coli* (Table 1). The other common character was the correlation of cell surface hydrophobicity and small colony variants or persister (metabolically inactive) or viable but not culturable population. This population increases with age of biofilm consortia. The old biofilm consortia (i.e. 48 to 96 hr) were found to be dominated by metabolically inactive and more hydrophobic cells (Table 1). These phenotypes were not recovered at pre-biofilm or at planktonic stage (Table 1 to 5). This phenotypic heterogeneity was a common character of *S. aureus* (100%) and *P. aeruginosa *(76.4%) biofilm consortia. However, in* E. coli *41.1% of biofilm consortia have shown this phenotypic heterogeneity. Viable plate count showed that CFU count dropped one logarithmic step after adopting biofilm mode of life in the entire subject isolates (Table 1). Population analysis assay showed that at pre-biofilm stage* E. coli *population dominated the multi-species biofilm consortia and consistently over-grew* P. aeruginosa and* *S. aureus *(Table 2). Results indicated that subject isolates of *S. aureus* and *E. coli* produced different amounts of biofilm but had similar timing of biofilm peaks and declines. Although, *P. aeruginosa *takes more time, comparatively, to enter from planktonic to biofilm mode of lifestyle, after adopting biofilm lifestyle* P. aeruginosa *showed dominance over* S. aureus *and* E. coli *(Table 2). Biofilm mass study by the crystal violet method indicates that average *P. aeruginosa* biomass was greater than that of *S. aureus *and* E. coli.* *P. aeruginosa* formed highly organized biofilms with inter-connected cells and continuous surface coverage (Figures 1a, b & c). Similar properties were observed in *S. aureus *monoculture biofilms (Figures 1f & g). Interestingly, after adaptation of biofilm mode of life, a morphological change in *P. aeruginosa* has been noticed (Figures 1b &c). The cells covered with extracellular matrix material showed a bricks like appearance. Conversely, *E. coli* showed scant biofilms with very small, discontinuous micro-colonies in monoculture and in co-culture conditions (Figures 1d & e). 

***Co-culture of S. aureus and P. aeruginosa***

Out of five, three co-cultured combinations showed biofilm formation. Surprisingly, *P. aeruginosa *dominated *S. aureus* at the pre-biofilm stage as well as in biofilm consortia (Table 4). Population analysis profile of biofilm consortia showed only *P. aeruginosa. *Moreover*, *when biofilm negative isolates were co-cultured, out of five only one showed biofilm formations and this was also dominated by *P. aeruginosa *(Table 4)*. * Co-culture of biofilm positive *P. aeruginosa* with biofilm negative *S. aureus *did not show any impact on *P. aeruginosa *biofilms. However, biofilm negative *P. aeruginosa *with biofilm positive *S. aureus* resulted in inhibition of *S. aureus* biofilm formation. Surprisingly, biofilm negative *P. aeruginosa *adopted biofilm mode of life after inhibition of *S. aureus* biofilm formation. On Congo-red agar, parallel inoculation of *P. aeruginosa *and biofilm positive *S. aureus *showed biofilm positive *S. aureus* growth without blackening. This suggests inhibition of polysaccharide production of *S. aureus*, which is a pre-requisite for *S. aureus* adhesion and biofilm formation (Table 2 & 4). 

**Figure 2:**
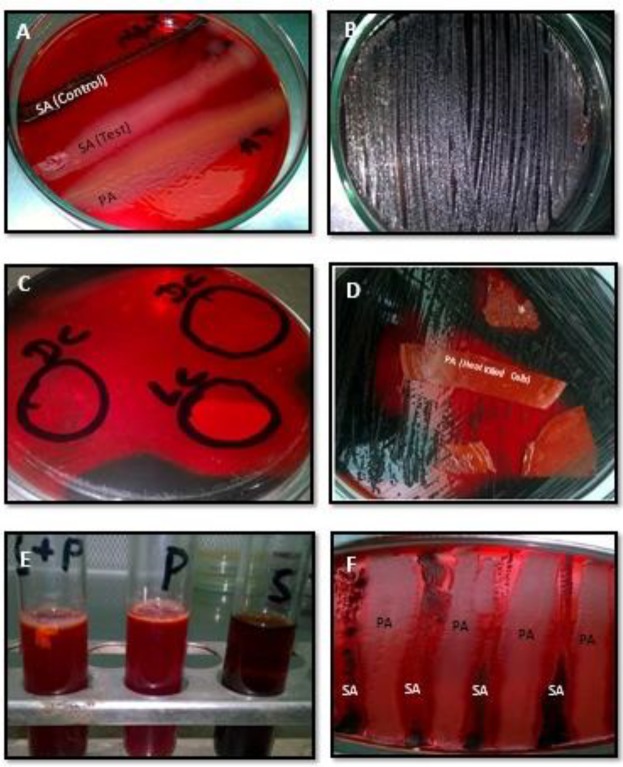
Biofilm inhibition Assay. (A & F) On Congo-red gar plate, parallel growth of (PA) *Pseudomonas aeruginosa *and (SA) *Staphylococcus aureus*, showed that *S. aureus *is unable to produce biofilm in the presence of *P. aeruginosa*. However, the control streaked away from *P. aeruginosa* showed blackening, which suggests biofilm formation. (B) Biofilm positive *S. aureus* on Congo-red agar (C) Application of (DC) dead cells and (LC) live cells of *P. aeruginosa* showed inhibition of biofilm formation of *S. aureus* on Congo-red agar. (D) Application of heat-killed cells of *P. aeruginosa* showed inhibition of biofilm formation of *S. aureus *on Congo-red agar. (E) In Congo-red broth *S. aureus* (S) was unable to adapt biofilm mode of growth in the presence of *P. aeruginosa* (P). P=*P. aeruginosa*, S=*S. aureus*. P+S=*P. aeruginosa + S. aureus*

**Figure 3:**
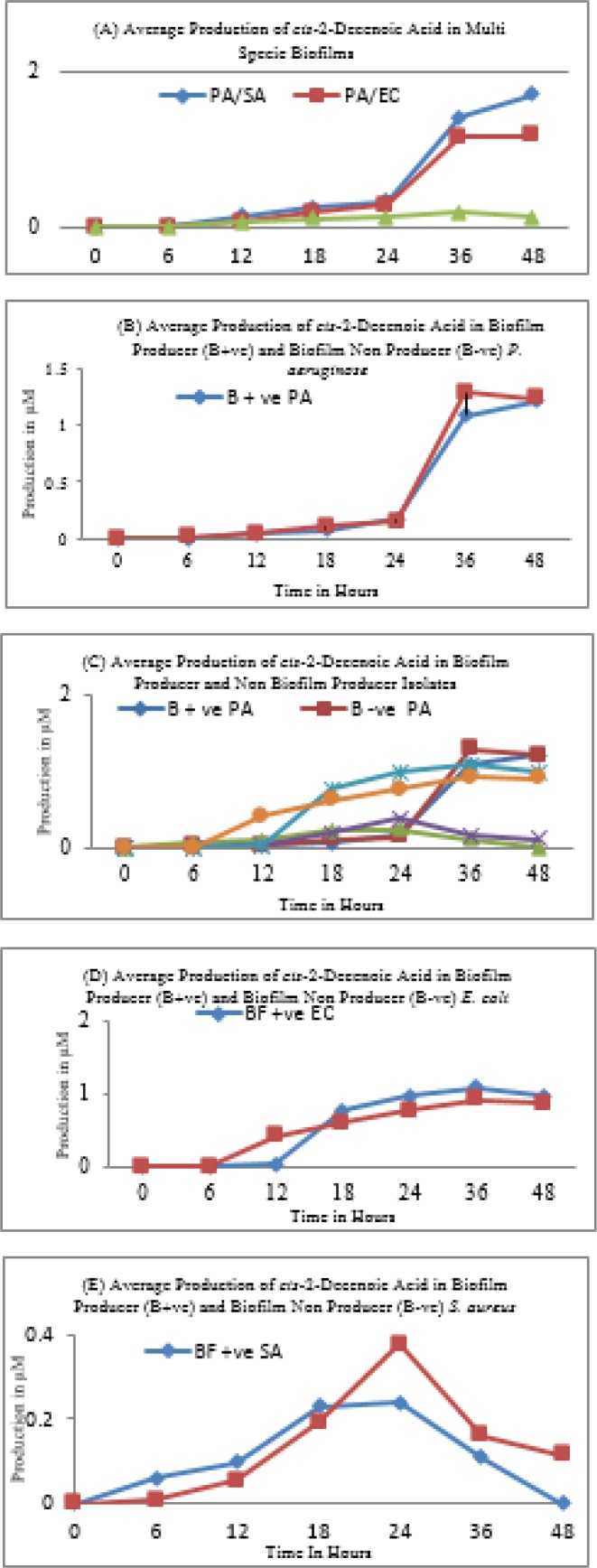
Production and impact of cis-2-Decenoic acid on biofilm. PA=* Pseudomonas aeruginosa*, SA=*Staphylococcus aureus*, EC=*Escherichia coli*

**Figure 4:**
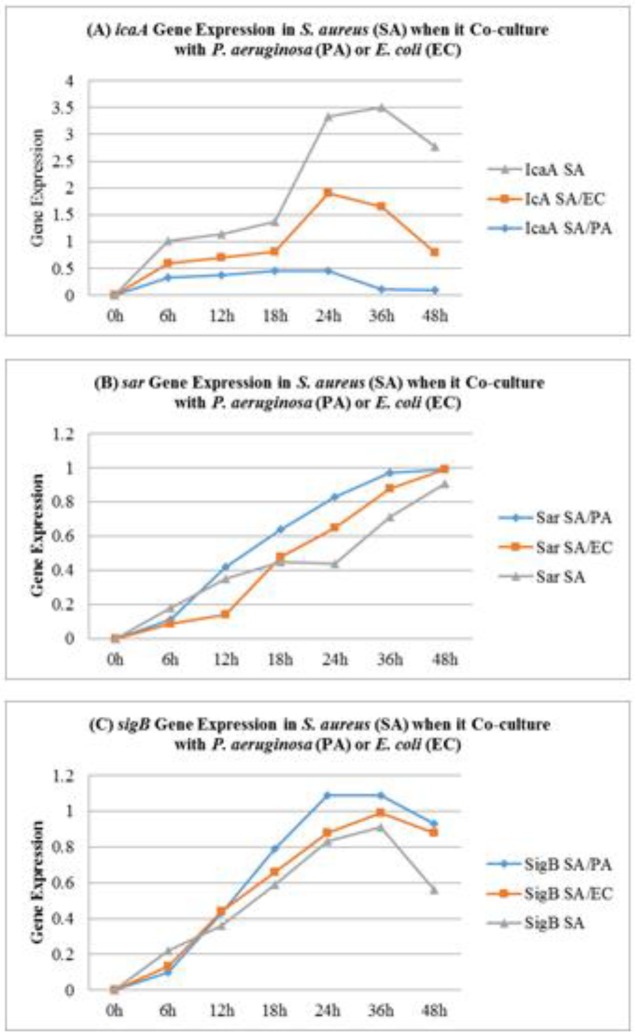
Impact of *Pseudomonas aeruginosa* (PA) and *Escherichia coli *on *icaA, sar *and *sigB* gene expression of *Staphylococcus aureus* (SA) in co-culture conditions

**Table 1:** Characterization of Mono-Specie Biofilms of *Pseudomonas aeruginosa *(P.A), *Escherichia coli *(E.C) and *Staphylococcus aureus *(S.A). Cell surface hydrophobicity, Planktonic population and SCVs or persister cells population at pre and post biofilm stage

Biofilm optical density (OD_578_^a^)								Cell surface hydrophobicity							Pre-biofilm population at 24 hr			Biofilm population at 48 hr			SCVs or persister cell population of biofilms		
*P.A*			*E.C*			*S.A*		Pre-Biofilm Stage at 24 hr				Biofilm Stage at 48 hr											
24 hr	48 hr	24 hr		48 hr	24 hr		48 hr		*P.A*	*E.C*	*S.A*	*P.A*	*E.C*	*S.A*	*P.A*	*E.C*	*S.A*	*P.A*	*E.C*	*S.A*	*P.A*	*E.C*	*S.A*
0.33	0.82	0.13		0.55	0.39		0.66		0.41	0.46	0.51	0.96	0.57	0.95	1x10^5^	1x10^5^	1x10^5^	1x10^4^	1x10^3^	1x10^3^	1x10^2^	<1cfu	1x10^1^
0.41	0.89	0.15		0.59	0.08		0.92		0.63	0.45	0.55	0.99	0.58	0.99	1x10^5^	1x10^6^	1x10^5^	1x10^3^	1x10^3^	1x10^3^	1x10^2^	<1cfu	1x10^1^
0.25	0.88	0.15		0.57	0.23		0.89		0.67	0.46	0.57	0.93	0.53	0.95	1x10^5^	1x10^6^	1x10^5^	1x10^3^	1x10^3^	1x10^4^	1x10^2^	<1cfu	1x10^3^
0.32	0.78	0.17		0.59	0.27		0.73		0.77	0.53	0.55	0.94	0.54	0.91	1x10^5^	1x10^6^	1x10^5^	1x10^3^	1x10^4^	1x10^4^	1x10^1^	<1cfu	1x10^3^
0.24	0.99	0.34		1.13	0.17		0.86		0.72	0.62	0.59	0.92	0.90	0.91	1x10^5^	1x10^6^	1x10^5^	1x10^3^	1x10^4^	1x10^4^	1x10^2^	1x10^1^	1x10^3^
0.42	1.11	0.33		0.98	0.21		0.60		0.73	0.61	0.63	0.91	0.85	0.99	1x10^5^	1x10^6^	1x10^5^	1x10^3^	1x10^3^	1x10^4^	1x10^2^	1x10^1^	1x10^3^
0.36	0.91	0.37		0.97	0.22		0.79		0.67	0.56	0.61	0.89	0.83	0.91	1x10^5^	1x10^5^	1x10^5^	1x10^3^	1x10^3^	1x10^4^	1x10^2^	<1cfu	1x10^2^
0.33	0.52	0.46		0.51	0.35		0.49		0.59	0.52	0.57	0.68	0.62	0.92	1x10^5^	1x10^5^	1x10^5^	1x10^3^	1x10^4^	1x10^4^	<1cfu	<1cfu	1x10^2^
0.39	0.88	0.37		0.73	0.33		0.45		0.62	0.51	0.68	0.90	0.77	0.91	1x10^5^	1x10^6^	1x10^5^	1x10^3^	1x10^4^	1x10^4^	1x10^2^	1x10^2^	1x10^2^
0.26	0.88	0.45		0.98	0.55		0.87		0.74	0.47	0.61	0.92	0.86	0.91	1x10^5^	1x10^5^	1x10^5^	1x10^3^	1x10^4^	1x10^3^	1x10^2^	1x10^2^	1x10^2^
0.34	0.56	0.44		0.63	0.54		0.81		0.77	0.43	0.45	0.85	0.64	0.59	1x10^5^	1x10^6^	1x10^5^	1x10^3^	1x10^4^	1x10^3^	1x10^2^	<1cfu	1x10^1^
0.27	0.79	0.49		1.07	0.55		0.86		0.72	0.62	0.35	0.87	0.81	0.55	1x10^5^	1x10^5^	1x10^5^	1x10^3^	1x10^3^	1x10^3^	<1cfu	<1cfu	1x10^1^
0.33	0.97	0.41		0.52	0.25		0.81		0.64	0.63	0.45	0.84	0.72	0.56	1x10^5^	1x10^6^	1x10^5^	1x10^3^	1x10^4^	1x10^4^	1x10^2^	<1cfu	1x10^1^
0.43	0.98	0.41		0.53	0.09		0.85		0.68	0.64	0.59	0.83	0.73	0.87	1x10^5^	1x10^6^	1x10^5^	1x10^3^	1x10^4^	1x10^3^	1x10^2^	1x10^1^	1x10^2^
0.41	0.95	0.47		0.55	0.08		0.97		0.66	0.66	0.51	0.94	0.71	0.97	1x10^5^	1x10^6^	1x10^5^	1x10^3^	1x10^4^	1x10^3^	<1cfu	1x10^2^	1x10^3^
0.32	0.62	0.45		1.04	0.23		0.52		0.49	0.64	0.44	0.76	0.91	0.99	1x10^5^	1x10^5^	1x10^5^	1x10^3^	1x10^3^	1x10^4^	<1cfu	1x10^2^	1x10^3^
0.33	0.61	0.49		1.04	0.27		0.79		0.43	0.65	0.47	0.73	0.88	0.82	1x10^5^	1x10^5^	1x10^5^	1x10^3^	1x10^3^	1x10^3^	1x10^2^	<1cfu	1x10^2^

**Table 2:** Characteristics of Multi-specie Biofilms of *Escherichia coli, Staphylococcus aureus *and *Pseudomonas aeruginosa*, Cell surface hydrophobicity, Planktonic population and SCVs or persister cells population at pre and post biofilm stage

Combinations	S #	Biofilm OD_578_^a^	Cell surface hydrophobicity			Pre-biofilm population after 18 hr incubation			Biofilm population after 48 hr of incubation			SCVs or persister cell population of biofilms		
			18 hr	24 hr	48 hr	*E. coli*	*S. aureus*	*P. aeruginosa*	*E. coli*	*S. aureus*	*P. aeruginosa*	*E. coli*	*S. aureus*	*P. aeruginosa*
Biofilm positive isolates in multi-specie biofilms	1	0.93	0.45	0.68	0.77	1x10^5^	1x10^4^	1x10^2^	1x10^2^	<10CFU	1x10^3^	1x10^1^	<10CFU	1x10^2^
	2	0.92	0.33	0.66	0.71	1x10^5^	1x10^4^	1x10^2^	1x10^2^	<10CFU	1x10^3^	1x10^1^	<10CFU	1x10^2^
	3	0.89	0.41	0.73	0.82	1x10^5^	1x10^4^	1x10^3^	1x10^2^	1x10^3^	1x10^3^	1x10^1^	<10CFU	1x10^2^
	4	0.97	0.44	0.77	0.87	1x10^5^	1x10^4^	1x10^3^	<10CFU	1x10^3^	1x10^3^	<10CFU	1x10^1^	1x10^2^
	5	0.99	0.22	0.71	0.89	1x10^4^	1x10^4^	1x10^3^	1x10^2^	<10CFU	1x10^3^	1x10^1^	<10CFU	1x10^2^
	6	1.01	0.24	0.73	0.94	1x10^5^	1x10^4^	1x10^3^	1x10^2^	<10CFU	1x10^3^	<10CFU	<10CFU	1x10^2^
	7	1.03	0.26	0.71	0.91	1x10^5^	1x10^5^	1x10^3^	<1CFU	1x10^3^	1x10^3^	<10CFU	<10CFU	1x10^2^
	8	0.92	0.34	0.67	0.77	1x10^4^	1x10^5^	1x10^3^	1x10^3^	<10CFU	1x10^3^	<10CFU	<10CFU	1x10^2^
	9	1.04	0.27	0.69	0.93	1x10^5^	1x10^4^	1x10^3^	1x10^3^	<10CFU	1x10^3^	<10CFU	<10CFU	1x10^2^
Biofilm Negative Isolates in multi-specie biofilms	1	BND	0.22	0.68	0.51	1x10^5^	1x10^4^	1x10^4^	BND	BND	BND	BND	BND	BND
	2	BND	0.19	0.66	0.52	1x10^5^	1x10^5^	1x10^4^	BND	BND	BND	BND	BND	BND
	3	0.86	0.33	0.69	0.72	1x10^5^	1x10^4^	1x10^3^	1x10^2^	<10CFU	1x10^3^	<10CFU	<10CFU	1x10^2^
	4	0.88	0.37	0.41	0.74	1x10^5^	1x10^4^	1x10^3^	1x10^2^	<10CFU	1x10^3^	1x10^1^	<10CFU	1x10^1^
	5	BND	0.31	0.44	0.46	1x10^5^	1x10^4^	1x10^4^	BND	BND	BND	BND	BND	BND
	6	BND	0.28	0.43	0.51	1x10^5^	1x10^4^	1x10^4^	BND	BND	BND	BND	BND	BND
	7	BND	0.27	0.42	0.55	1x10^5^	1x10^4^	1x10^4^	BND	BND	BND	BND	BND	BND
	8	BND	0.22	0.34	0.52	1x10^5^	1x10^4^	1x10^4^	BND	BND	BND	BND	BND	BND
	9	BND	0.18	0.33	0.47	1x10^5^	1x10^4^	1x10^3^	BND	BND	BND	BND	BND	BND

**Table 3:** Characteristics of duel specie biofilms of *Escherichia coli *and *Staphylococcus aureus *Cell surface hydrophobicity, Planktonic population and SCVs or persister cells population at pre and post biofilm stage

Combination	S #	Biofilm OD_578_^a^	Cell surface hydrophobicity			Pre-biofilm population after 24 hr of incubation		Biofilm population after 48 hr of incubation		SCVs or persister cell population of biofilms	
			At 18 hr	At 24 hr	At 48 hr	*E. coli*	*S. aureus*	*E. coli*	*S. aureus*	*E. coli*	*S. aureus*
Biofilm positive Isolates	1	0.89	0.33	0.61	0.77	1x10^5^	1x10^4^	1x10^2^	1x10^3^	1x10^1^	1x10^2^
	2	0.83	0.41	0.63	0.74	1x10^5^	1x10^4^	1x10^1^	1x10^3^	1x10^1^	1x10^2^
	3	0.92	0.44	0.56	0.82	1x10^5^	1x10^4^	1x10^2^	1x10^2^	<10CFU	1x10^2^
	4	0.99	0.42	0.55	0.93	1x10^5^	1x10^5^	1x10^2^	1x10^2^	<10CFU	1x10^3^
	5	0.93	0.26	0.62	0.87	1x10^4^	1x10^4^	1x10^2^	1x10^2^	<10CFU	1x10^2^
Biofilm negative isolates	1	BND	0.22	0.33	0.51	1x10^5^	1x10^5^	BND	BND	BND	BND
	2	BND	0.18	0.21	0.44	1x10^5^	1x10^4^	BND	BND	BND	BND
	3	BND	0.17	0.22	0.28	1x10^5^	1x10^4^	BND	BND	BND	BND
	4	BND	0.21	0.23	0.27	1x10^5^	1x10^4^	BND	BND	BND	BND
	5	BND	0.23	0.34	0.47	1x10^5^	1x10^5^	BND	BND	BND	BND
Biofilm positive *S. aureus* with biofilm negative *E. coli*	1	0.66	0.22	0.49	0.73	1x10^5^	1x10^4^	<10CFU	1x10^3^	<10CFU	1x10^2^
	2	0.72	0.21	0.44	0.72	1x10^5^	1x10^4^	<10CFU	1x10^3^	<10CFU	1x10^1^
	3	0.81	0.19	0.47	0.71	1x10^5^	1x10^4^	1x10^1^	1x10^3^	<10CFU	1x10^1^
	4	0.79	0.32	0.66	0.69	1x10^5^	1x10^4^	1x10^1^	1x10^3^	<10CFU	1x10^1^
	5	0.77	0.33	0.61	0.69	1x10^5^	1x10^4^	<1CFU	1x10^3^	<10CFU	1x10^1^
Biofilm negative *S. aureus* with biofilm positive *E. coli*	1	0.66	0.45	0.59	0.77	1x10^5^	1x10^4^	1x10^2^	1x10^3^	1x10^1^	<10CFU
	2	0.71	0.43	0.61	0.78	1x10^5^	1x10^4^	1x10^2^	<10CFU	<10CFU	<10CFU
	3	0.73	0.42	0.63	0.76	1x10^4^	1x10^4^	1x10^3^	<10CFU	<10CFU	<10CFU
	4	0.77	0.41	0.62	0.69	1x10^5^	1x10^4^	1x10^3^	1x10^1^	<10CFU	<10CFU
	5	0.69	0.39	0.64	0.71	1x10^5^	1x10^4^	1x10^2^	1x10^1^	<10CFU	<10CFU

**Table 4:** Characteristics of Duo-Specie Biofilms of *Pseudomonas aeruginosa *and *Staphylococcus aureus *Cell surface hydrophobicity, Planktonic population and SCVs or persister cells population at pre and post biofilm stage

Combination	S #	Biofilm OD_578_^a^	Cell surface hydrophobicity			Pre-biofilm population after 24 hr of incubation		Biofilm population after 48 hr of incubation		SCVs or persister cell population of biofilms	
			At 18 hr	At 24 hr	At 48 hr	*P.* *aeruginosa*	*S. aureus*	*P.* *aeruginosa*	*S. aureus*	*P.* *aeruginosa*	*S. aureus*
Biofilm positive isolates	1	0.97	0.23	0.49	0.88	1x10^4^	1x10^5^	1x10^3^	1x10^1^	1x10^2^	<10CFU
	2	0.99	0.21	0.63	0.87	1x10^4^	1x10^5^	1x10^3^	<10CFU	1x10^2^	<10CFU
	3	0.93	0.21	0.47	0.81	1x10^4^	1x10^5^	1x10^3^	<10CFU	1x10^2^	<10CFU
	4	0.98	0.28	0.57	0.88	1x10^4^	1x10^4^	1x10^2^	<10CFU	1x10^2^	<10CFU
	5	0.97	0.31	0.61	0.82	1x10^4^	1x10^5^	1x10^2^	<10CFU	1x10^2^	<10CFU
Biofilm negative isolates	1	BND	0.19	0.27	0.28	1x10^4^	1x10^5^	BND	BND	BND	BND
	2	BND	0.18	0.27	0.31	1x10^4^	1x10^5^	BND	BND	BND	BND
	3	0.61	0.31	0.39	0.57	1x10^4^	1x10^5^	1x10^2^	<10CFU	<10CFU	<1CFU
	4	BND	0.13	0.21	0.25	1x10^4^	1x10^5^	BND	BND	BND	BND
	5	BND	0.21	0.19	0.35	1x10^4^	1x10^5^	BND	BND	BND	BND
Biofilm positive *S. aureus* with biofilm negative *P.* *aeruginosa*	1	BND	0.19	0.26	0.33	1x10^5^	1X10^3^	BND	BND	BND	BND
	2	BND	0.21	0.28	0.31	1x10^4^	1X10^4^	BND	BND	BND	BND
	3	0.58	0.26	0.47	0.66	1x10^5^	1X10^4^	1x10^3^	<10CFU	<10CFU	<10CFU
	4	0.66	0.19	0.49	0.73	1x10^5^	1X10^3^	1x10^3^	<10CFU	<10CFU	<10CFU
	5	0.63	0.23	0.53	0.79	1x10^4^	1X10^3^	1x10^3^	<10CFU	<10CFU	<10CFU
Biofilm negative *S. aureus* with biofilm positive *P.* *aeruginosa*	1	0.66	0.22	0.56	0.77	1x10^4^	1X10^4^	1X10^3^	<10CFU	1x10^2^	<10CFU
	2	0.81	0.22	0.49	0.81	1x10^4^	1X10^4^	1X10^3^	<10CFU	1x10^2^	<10CFU
	3	0.89	0.21	0.53	0.83	1x10^4^	1X10^4^	1X10^3^	<10CFU	<10CFU	<10CFU
	4	0.82	0.23	0.52	0.84	1x10^4^	1X10^4^	1X10^3^	1x10^2^	1x10^2^	<10CFU
	5	0.88	0.25	0.55	0.82	1x10^4^	1X10^4^	1X10^3^	1x10^2^	1x10^2^	<10CFU

***Co-culture of S. aureus with E. coli***

Co-culture of *S. aureus* with *E. coli* showed that the *E. coli* dominated pre-biofilm stage (Table 2 & 3). However, in biofilm consortia, *S. aureus* overcame *E. coli *and occupied more space. Like *P. aeruginosa, S. aureus *also showed organized and inter-connected cells. The aged biofilm consortia was also dominated by SCVs of *S. aureus* (Tables 2 & 3). 

***Co-culture of P. aeruginosa with E. coli***

In this combination, it was again noticed that *E. coli* dominated pre-biofilm stage (Table 5). However, after adoption of biofilm mode of life P. aeruginosa took over E. coli and slowly dominated the whole consortium. Although *P. aeruginosa* adopted biofilm mode of life later than *E. coli*, slowly it took over and occupied more space (Table 5). 

***Biofilm inhibition assay and cis-2-decenoic acid production***

Supernatants of all of the subject isolates of *P. aeruginosa* had a pronounced effect on the *S. aureus *and *E. coli *at the pre-biofilm stage. All biofilm producing isolates of *S. aureus* and *E. coli* were unable to adopt biofilm mode of life in the presence of cell-free supernatants of *P. aeruginosa* (Figures 2a, c, d, e & f)*. *A similar inhibitory effect was noticed when heat-killed dead cells of *P. aeruginosa *were applied. Moreover, this cell-free extract was comparatively ineffective against established biofilms of *S. aureus*. Interestingly in established biofilms, the small colony variants of *S. aureus* were not affected by the presence of heat-killed cells or cell-free extract of *P. aeruginosa *and persisted for a long time and remained adherent. Contrary to this, cell-free extract of *P. aeruginosa *dispersed established biofilms of *E. coli *by killing or lysis of target cells. The results of Gas chromatography and mass spectrometry (GC-MS) revealed that cell-free extract of *P. aeruginosa* contains a sustainable amount of cis-2-decenoic acid (Figure 3). Comparative analysis showed that biofilm non-producer isolates produce more cis-2-decenoic acid and the highest production was achieved at late log phase or stationary phase (Figures 3b, d & e). The highest production of cis-2-decenoic was noticed when *P. aeruginosa *and *S. aureus* were grown in combination (Figure 3a). Similarly, *E. coli* and *P. aeruginosa* showed a higher quantity of cis-2-decenoic as compared to the *E. coli* and *S. aureus* combination (Figure 3a). Moreover, highest quantity of extracellular cis-2-decenoic fatty acid was recovered from *P. aeruginosa *and lowest quantity was recovered from *S. aureus *(Figure 3a)*.* Moreover, cis-2-decenoic non-producer *P. aeruginosa* has no impact on *E. coli* and *S. aureus* growth either in planktonic or biofilm stage. 

***S. aureus icaA, Sar, and SigB gene expression in Co-culture conditions***

In co-culture with *P. aeruginosa*, a significant reduction was noticed in the *icaA* gene, which is considered to be responsible for biofilm formation in* S. aureus. *The *icaA *ene was drastically influenced by the presence of *P. aeruginosa *and >10 fold reduction was noticed in the expression of this gene by RT-PCR. The presence of *E. coli* also influences the *icaA* gene expression in *S. aureus.* However, *S. aureus* isolates survived in the presence of *P. aeruginosa* and exhibited the highest activity of *sigB* and *sarA. *Similarly, the presence of *E. coli* also augments *sigB* and *sarA* gene expression in *S. aureus* (Figures 4a to 4c)*.*

## Discussion

In nature, competition between bacterial strains and species appears to be a common phenomenon. According to a study (34), bacteria appear to attack back when they are attacked. It has been reported that stress factors, e.g. nutrient depletion, overcrowding, and invaders, induce bacteria to release their own antibiotics or toxins for self-defense (35). These antibiotics or toxins may also work as a signaling molecule for other bacteria (36). Consequently, other species or genera residing in the same niche become alert and defend themselves, sometimes by adopting the biofilm mode of life (37). It is well known that most of the antibiotics or other antibacterial molecules are unable to penetrate in biofilm consortia (38, 39). In a natural environment, biofilms mostly consist of complex and multiple communities (1). In this study, we attempted to explore multi-species biofilm formation, biofilm dispersion, and planktonic interaction in *P. aeruginosa*, *S. aureus,* and *E. coli* in a single assay. It has been noticed that at the pre-biofilm stage, the entire range of subject isolates exhibited hydrophilic surface properties. It suggests that this is a common character of monoculture as well as co-culture. However, after the adoption of biofilm mode of life, the surface properties of subject isolates were changed from hydrophilic to hydrophobic. According to Kochkodan *et al.* (40) and Giaouris *et al.* (41), cell surface hydrophobicity is a major factor for bacterial adhesion and biofilm formation. This is supported by Krasowska and Sigler (42). The other significant character of the subject isolates of *P. aeruginosa* and *S. aureus* is the appearance of metabolically inactive phenotypes i.e. small colony variants SCVs. These phenotypes dominate the biofilm consortia both in monoculture and co-culture state. The SCVs takeover metabolically active population as biofilm consortia becomes older. Interestingly, the hydro-phobicity of biofilm consortium increases as the population of SCVs increases. These characters were the common property of *S. aureus* and *P. aeruginosa* and in agreement with our previous study of *S. aureus* and MRSA biofilms (43). According to Kahl *et al*. (44), although the SCVs are metabolically inactive and most of the genes are non-functional but genes for biofilm formation and adhesion are up-regulated. Contrary to this, our study showed that *icaA*, *sigB* and *sarA* gene expression was more reduced or even arrested in SCVs of *S. aureus* (45). Interestingly, in present study, *P. aeruginosa* seems to augment *sarA* and *sigB* expression and diminish *icaA* gene expression in co-cultured isolates of *S. aureus*. The same phenomenon was noticed when *S. aureus *was co-cultured with *E. coli*. It has been noticed that in biofilm consortia, majority of *S. aureus* were dispersed or killed by the presence of *P. aeruginosa* and only minor population was able to survive. These survivors seem to adopt ica independent biofilm mode of life because the icaA gene expression was reduced or arrested in the presence of P. aeruginosa or *E. coli*. This was confirmed by the *sarA* and *sigB* gene expression studies. It is reported that *sarA* is significant for *S. aureus* biofilm development (46). These results indicate that in the conditions where *icaA* gene is absent or non-functional, *sarA* mediates biofilm formation in Staphylococci. Similarly, *sigB* plays a crucial role in the survival of *S. aureus* in stress environment (47). In a recent study, Tuchscherr *et al*. (47), reported that *sigB* enables *S. aureus *to switch from planktonic to small colony variants. It is reported that SCVs have a major role in stability and persistence of *S. aureus *and *P. aeruginosa* biofilms (45, 48). Comparative analysis of co-culture population showed that *E. coli* dominated pre-biofilm population and adopted biofilm mode of life before *S. aureus* and *P. aeruginosa*. This might be due to comparatively shorter generation time of *E. coli* and weaker competitive nature of *S. aureus*. Accordingly, Culotti and Packman (14) reported that co-culturing of *P. aeruginosa *augment the ability of *E. coli* to persist and grow in aquatic environment. Chu *et al*. (49) suggested that indole produced by *E. coli* arrests the quorum singling program and other virulence programs of *P. aeruginosa*. This supports *E. coli* to survive and dominate the *P. aeruginosa* and *S. aureus* in co-culture environment. Contrary to this, Sadowska *et al*. (50) reported that co-culturing of *E. coli *has no effect on the growth of *S. aureus*. This scenario depicted the dominance of *E. coli *at planktonic stage. However, once *E. coli* population reaches the decline stages and is unable to produce extracellular metabolites, *P. aeruginosa* and *S. aureus* proliferate and take advantage. Furthermore, after adoption of biofilm state *S. aureus* and *P. aeruginosa* occupy more space and disperse *E. coli *from biofilm consortia. It was noticed that as *P. aeruginosa* population increases in biofilm consortia the population of *E. coli* decreases. Same phenomenon was noticed for *P. aeruginosa* against *S. aureus*. This might be due to cell surface hydrophobicity. It was noticed that more hydrophobicity produces strong biofilms, persist for a long time and difficult to disperse (43). Cell surface hydrophobicity assays revealed that *S. aureus* and *P. aeruginosa* were more hydrophobic as compared to *E. coli*. The other property of *S. aureus* and *P. aeruginosa* which help them to persist for long time is the switch to small colony variants. These metabolically inactive phenotypes persist for long time and increases survival of *S. aureus* and *P. aeruginosa* under stress conditions (51). On the other hand, *E. coli* are unable to switch to small colony variants, so they are unable to withstand toxic effect of antibacterial metabolites of *S. aureus* or *P. aeruginosa* and are gradually dispersed from co-cultured consortia. Moreover, the findings of Estrela *et al*. (52) and Davies and Marques (53) suggested that *P. aeruginosa* producing diffusible signaling molecules e.g. *cis*-2-dodecenoic acid and *cis*-2-decenoic acid which are found to be effective in biofilm dispersion of not only *P. aeruginosa*, but also *Streptococcus mutans*, *E. coli*, *K. pneumoniae*, *Proteus mirabilis*, *Streptococcus pyogenes*, *Bacillus subtilis*, *S. aureus*, and the yeast, *Candida albicans*. In the present study, it was noticed that *P. aeruginosa* produces *cis*-2-decanoic acid and the production of this compound increases in co-culture state either with *S. aureus *or *E. coli*. This suggests that strong competitive behavior and dominance of *P. aeruginosa*. Consequently, *E. coli* and *S. aureus* are unable to adopt biofilm mode of life in the presence of *P. aeruginosa*. Similarly, *P. aeruginosa *occupy more space and it disperses *S. aureus* population when it grows with *S. aureus*. This is also supported by the findings of Filkins *et al.* (10) and Kim *et al*. (54). They have suggested that in co-culture *P. aeruginosa* reduces *S. aureus* viability by producing 2-heptyl-4-hydroxyquinoline N-oxide (HQNO) and siderophores (10). In three species biofilms, the *P. aeruginosa* dominate the consortia. Although, *E. coli *dominates the pre-biofilm stage but is unable to proliferate in three species biofilms, perhaps due to the presence of *P. aeruginosa,* which was evidently harmful for other members of three species biofilm consortia. This antagonistic effect is probably caused by extracellular metabolites of *P. aeruginosa* e.g. *cis*-2-decanoic acid. Moreover, cell-free extract as well as dead cells of *P. aeruginosa* were found to inhibit *S. aureus* and *E. coli* biofilm formation and also disperse pre-established biofilms. This suggests the antagonistic or antibacterial nature of compounds of *P. aeruginosa*. As we used heat-killed cells in this study, it suggests this compound is cell bound and heat stable. On the other hand cell-free extract also disperse biofilm indicating the extracellular activity of *P. aeruginosa*. Accordingly, Qin *et al*. (55) suggested that *P. aeruginosa* extracellular products are important microbial competition factors that overcome and disperse Staphylococcal biofilms. However, *S. aureus* or *E. coli* cell-free culture extract had no effect on *P. aeruginosa* or on each other’s biofilms or on planktonic growth. 

## Conclusion

The present study indicates that *P. aeruginosa* and *S. aureus* exhibited hydrophobic surface properties and are comparatively slow growing. Whereas subject isolates of *E. coli* exhibited hydrophilic surface properties and were comparatively fast growing. Due to hydrophobic surface properties and slow growth rate, *P. aeruginosa* and *S. aureus* showed strong biofilm formation and persistence. Among all three subject organisms, *P. aeruginosa* is comparatively more hydrophobic, slow growing, and adopts biofilm mode of life pretty late; in this category, *S. aureus* comes next. Moreover, other hydrophobic isolates, e.g. *P. aeruginosa* and *S. aureus* are more likely to switch to small colony variants. Biofilm consortia dominated with small colony variants persist for a long time and are difficult to disperse. With these properties, *P. aeruginosa* dominates and antagonizes other counterparts in biofilm consortia and also produces *cis*-2-decanoic acid to inhibit or disperse biofilms of other organisms. Although, majorities of the *S. aureus* were unable to compete with *P. aeruginosa*, however, a minor population did compete, survive, and persist in biofilm consortia as small colony variants. The survivors showed higher expression of *sigB* and *sarA* genes. These results suggest that cell surface hydrophobicity is directly proportional to biofilm formation and SCVs population and inversely proportional to the growth rate of the subject isolates. 
